# Contrasting temperature responses of dissolved organic carbon and phenols leached from soils

**DOI:** 10.1007/s11104-015-2678-z

**Published:** 2015-09-23

**Authors:** Jonathan S. Williams, Jennifer A. J. Dungait, Roland Bol, Geoffrey D. Abbott

**Affiliations:** School of Civil Engineering and Geosciences, Drummond Building, Newcastle University, Newcastle-upon-Tyne, NE1 7RU UK; Department of Sustainable Soils and Grassland Systems, Rothamsted Research, North Wyke, Okehampton, Devon EX20 2SB UK; Institute of Bio- and Geosciences, IBG-3: Agrosphere, Forschungszentrum Jülich GmbH, 52425 Jülich, Germany

**Keywords:** Dissolved organic carbon, Leaf litter, Phenols, Seasonal temperature, Grassland soil

## Abstract

**Background and aims:**

Plant-derived phenols are a major input to the terrestrial carbon cycle that might be expected to contribute substantially to dissolved organic carbon (DOC) losses from soils. This study investigated changes in DOC and phenols in leachates from soil treated with individual plant litter types under seasonal temperature change.

**Methods:**

Senescing grass, buttercup, ash and oak litters were applied to soil lysimeters. Leachates were collected over 22 months and analysed for DOC and phenols. Phenols in litter and DOC were analysed using on-line thermally assisted hydrolysis and methylation with tetramethylammonium hydroxide (TMAH).

**Results:**

Mass loss differed between litter type (buttercup>ash>grass>oak). Phenol concentrations in the senescing litters (<2 % TOC) were small, resulting in minor losses to water. Seasonal soil temperature positively correlated with DOC loss from litter-free soils. An initial correlation between temperature change and total phenol concentration in grass and ash litter treatment leachates diminished with time. Dissolved phenol variety in all litter-amended soil leachates increased with time.

**Conclusions:**

Plant-derived phenols from senescing litter made a minor contribution to DOC loss from soils. The strength of the relationship between seasonal temperature change and phenol type and abundance in DOC changed with time and was influenced by litter type.

**Electronic supplementary material:**

The online version of this article (doi:10.1007/s11104-015-2678-z) contains supplementary material, which is available to authorized users.

## Introduction

Losses of dissolved organic carbon (DOC) from soils are amongst the least understood fluxes of the terrestrial carbon cycle. There is an economic and environmental imperative to understand and manage DOC inputs to improve water quality (Collins et al. 2013), and by the wider ambition to prevent soil organic carbon (SOC) losses to improve soil quality and to mitigate greenhouse gas emissions (Lal 2004). The transfer of organic carbon from plants to soil to waterways is governed by direct surface flows of particulate organic matter (POM) and sediment-associated organic carbon (Peukert et al. 2014), and sub-surface flows of DOC via hydrological pathways through the soil profile (Lloyd et al. 2012). The latter pathway is the most difficult to quantify because of the difficulty in meaningfully intercepting flows for measurement, and the general lack of knowledge about the factors that control the rates of turnover and loss of SOC as DOC (see recent review by Kaiser and Kalbitz 2012).

Understanding the temperature dependence of the rates of SOC processes is fundamental to determining the effect of global warming on soil C storage (Bol et al. 2003). The magnitude of change in the rate of SOC decomposition as temperature increases by 10 Kelvin (K) is described by the Q10 parameter (Davidson and Janssens 2006). However, experimental warming of soils has been observed to increase or decrease the rate of SOC losses measured as CO_2_ efflux (Karhu et al. 2014). Moreover, the relationship between temperature variation and losses of SOC by leaching are less well explored. A correlation between increasing decadal mean summer temperatures and increased UK riverine DOC concentrations was reported by Worrall et al. (2003), but Benner and Kaiser (2011) observed larger DOC and lignin phenol concentrations in river water in winter compared with late spring.

The temperature sensitivity of the rate of decomposition is assumed to increase as substrate quality decreases (where quality is defined as ‘the number of enzymatic steps required to release as CO_2_ a carbon atom from an organic compound’; Bosatta and Agren 1999). This phenomenon has been reported for forest soils (Bol et al. 2003; Waldrop and Firestone 2004; Kalbitz et al. 2007), grassland soils (Conant et al. 2008) and cultivated soils (Hartley and Ineson 2008), although Liski et al. (1999) suggested that the decomposition of old SOC in a boreal forest soil was resistant to temperature change. The chemical identity of the old ‘stable’ SOC pool has been sought in order to increase carbon sequestration by managing inputs of inherently recalcitrant organic matter, e.g. Lorenz et al. (2007). However, the idea that the resistance of SOC to decomposition can be based solely on its chemical structure has been disputed in favour of the wider role of environmental conditions such as soil moisture and temperature, and accessibility to decomposer organisms and/or their enzymes (Schmidt et al. 2011; Dungait et al. 2012) including stabilisation by organomineral interactions (Bol et al. 2009).

The variability in the rates of decomposition of different leaf litters correlates strongly with the physical and chemical characteristics of green leaves that persist through senescence (Cornwell et al. 2008). Phenols released from plant litter during decomposition derive from a range of protective biopolymers amongst which lignin is predominant, and tannins may also contribute substantially to tree leaf litter (Nierop and Filley 2007). Plant litter with relatively high lignin content is often described as having poor substrate quality (Grandy and Neff 2008). However, substantial lignin decomposition occurs in the initial stages of leaf decomposition (Klotzbücher et al. 2011a,b) and unmodified lignin is rarely detected in surface soil horizons (except in recognisable POM) where 48–87 % of the initial lignin content in buried litter bags is degraded within 5 years (Thevenot et al. 2010). Indeed, contrasting responses of lignin decomposition in soils to temperature increases have been reported, ranging from an increase (Pisani et al. 2015), to no change (Zhang et al. 2011), to a decrease (Amelung et al. 1999). Austin et al. (2014) concluded that the lignin concentration of plant litters is not sufficient to explain the mechanistic patterns of litter decomposition in terrestrial ecosystems. Furthermore, lignin degradation has been shown to be monomer specific (Bahri et al. 2006; Dungait et al. 2008) and the transport of DOC through the soil may fractionate lignin degradation products through the processes of adsorption and desorption (Hernes et al. 2007) which are affected by temperature (Conant et al. 2011). Less is known about the fate of tannins which are a diverse group of secondary compounds that can hinder rates of decomposition by complexing with proteins (Kraus et al. 2003). As a consequence, uncertainty remains regarding the importance of the contribution of plant-derived phenols in general to DOC losses from litter to soils to waterways, and how abiotic factors including temperature change affect its rate of loss.

In this study, we tested the overall hypothesis that the release of phenols from plant litters from soils as a component of DOC is controlled by seasonal changes in temperature. This was investigated by allowing plant litter from contrasting vegetation types typical of local land uses in SW England to decompose naturally on soil lysimeters under controlled irrigation and exposed to near-to-natural temperature variation for 22 months. Phenol degradation in the litters and losses in leachates was analysed on-line using thermally assisted hydrolysis and methylation (THM) in the presence of tetramethylammonium hydroxide (TMAH).

## Materials and methods

### Experimental design

A well-drained, coarse, loamy soil (Rivington series; Eutric Endoleptic Cambisol; Landis 2015); 0–23 cm depth, from permanent pasture at Bicton College (East Budleigh, Devon, UK, SY070865) was sieved (6.35 mm) to homogenise soil chemistry (pH 7.3) and remove large stones and plant residues. Twenty lysimeters were packed with 6.4 L sieved soil at optimum soil-water content (17.2 %) and bulk density (ρb = 1.73 g cm^−3^) and suspended on frames 300 mm above the ground to allow leachate collection and avoid a temperature gradient. Aluminium rings (255 mm diameter, 127 mm height) were pressed into the lysimeter soil surfaces to a depth of approximately 10 mm to prevent boundary flow between the soil core and inner lysimeter wall. Three temperature data loggers (Tinytag) connected to sensors (PB-5002-IM5 10K NTC, www.tinytag.info) were inserted into the soil of three different lysimeters at 3 cm depth and programmed to record soil temperature every 30 min.

‘Brown’ (senescing) plant litter was collected from different land use types at Rothamsted Research at North Wyke in Devon, UK (SX650995) in December 2009 to ensure that the litters were colonised by the natural litter decomposers for each location and stored at 4 °C until use. Grass litter was collected from agricultural grassland and divided into its dominant monocot grass (*Lolium perenne* and *Holcus lanatus*) and dicot buttercup (*Ranunculus repens*) species. Ash (*Fraxinus excelsior*) leaf litter was sampled from the floor of pure stands in managed agroforestry plots, and oak (*Quercus robur*) leaves were collected from the floor of unmanaged native woodland. Plant litter was applied to the soil surface of each of the 20 lysimeters to supply the same TOC (%) per m^2^ (Table 2) in May 2010. The five treatments (four vegetation types plus no vegetation (control)) were applied at random to lysimeters by using a random number generator. The lysimeters were irrigated for 15 s with tap water (pH 7.7) every 7 days using nozzles mounted 10 cm above each lysimeter to maintain soil moisture. The nozzle mean flow rate for each lysimeter was 275 mL/min to provide an even coverage of water to the vegetation litter surface.

### Leachate sampling

Leachate samples were collected after 82 (August), 143 (October), 200 (December), 263 (February), 381 (June), 459 (August) and 671 (March) days. On each occasion, 2300 mL tap water was applied to the top of each lysimeter and the water allowed to drain for 2 h into weighed amber glass bottles (Fisher Scientific, part no. FB73180). The volume of water was calculated using the lysimeter soil pore volume (2246 cm^3^) to create piston-flow. The pH of the leachate was determined for a 10 mL sub-sample (Supplementary Table 1). The leachates were acidified to pH 2 (trace analysis grade HCl acid, 37 %, Fisher Scientific). A subsample (10 mL) was analysed immediately for total organic carbon (TOC) concentration (mg L^−1^) using a TOC analyser (CA14 Formacs, Skalar (UK) Ltd). The carrier gas was purified air, supplied by a TOC gas generator (scrubbed of CO_2_ and moisture), and the inorganic catalyst solution was 2 % orthophosphoric acid.

The remainder of the leachate was extracted using solid phase extraction (SPE) using a modified version of the method described by Louchouarn et al. (2000). Reverse phase C_18_ end capped solid phase extraction (SPE) cartridges (60 mL, 10 g, Mega-Bond Elut, Agilent Technologies) were mounted on a vacuum manifold (VAC ELUT-20, 13 × 75 mm, Varian) connected to a vacuum pump (Gast Diaphragm pump, model: DOA-P504-BN. Gast Manufacturing, Inc. U.S.A.) via a liquid trap (Carboy Bottle 20 L, part 2226-0050 with filling venting closure, part 2161-0830, Varian Ltd.). Each cartridge was preconditioned with 100 mL methanol (HPLC grade, Fisher Scientific) followed by 50 mL pure water (MilliQ Gradient A10) acidified to pH 2 (Trace analysis grade HCl acid, 37 %, Fisher Scientific). Leachate samples (approx. 2 L) were drawn through the SPE cartridges at an average flow rate of approximately 20 mL min^−1^ via a Teflon transfer pipe (1/8 in. × 0.1 in. Part AL20096, Varian Ltd.) and adapters (part no. 12131004, Varian Ltd.). The cartridges were rinsed with 50 mL pure water (pH 2) to remove any residual salts. Collection bottles (60 mL, Part BTF-543-030X, Fisher Scientific) were placed inside the vacuum manifold under each SPE cartridge prior to eluting the organic phase containing phenols with 50 mL methanol. The methanol was evaporated from the collection bottles at 40 °C under a stream of N_2_ leaving the DOC residue for analysis.

### Sample analysis

Dried and ground litter, soil and DOC residues were characterised for total organic C (TOC) and total N (TN) contents using a NA2000 analyser (Carlo Erba Instruments, Wigan, UK) and a 20–22 isotope ratio mass spectrometer (SerCon Ltd., Crewe, UK).

Individual phenols in litter, soil and DOC were directly analysed using thermally assisted hydrolysis and methylation (THM) with tetramethylammonium hydroxide (TMAH) using on-line pyrolysis gas chromatography mass spectrometry (Py-GC-MS). Samples were weighed (0.5 to 1.5 mg) into quartz pyrolysis tubes plugged with solvent-extracted glass wool. 5α-androstane in dichloromethane (3 μL; 0.1 mg/mL) was added as an internal standard to each pyrolysis tube. Immediately prior to analysis, 5 μL of an aqueous solution of TMAH (25 %, *w*/*w*) was added to the sample. The tube was inserted into the platinum pyrolysis coil of a pyroprobe (CDS Pyroprobe 1000, CDS Analytical Inc.) and flash pyrolysed at 610 °C for 10 s (20 °C ms^−1^ temperature ramp (Abbott et al. 2013). The pyroprobe interface (CDS 1500 valved interface, CDS Analytical Inc.) was maintained at 340 °C with the pyrolysis products passing into an HP6890 gas chromatograph (GC) with an open split (30 mL/min) and a 60 m HP5-MS column (0.25 mm internal diameter, 0.25 μm film thickness, J&W Scientific, USA). Helium was used as the carrier gas at a flow rate of 1 mL/min. The GC oven was programmed from 50 to 220 °C at a rate of 1.5 °C/min, and then held isothermally for 1 min, and then raised to a final temperature of 320 °C at a rate of 15 °C/min and held isothermally for 16 min. Product detection was carried out using an HP5973 series mass selective detector in full scan mode (m/z 50 to 700). Compound identification was based on the NIST98 mass spectral library, literature (Vane et al. 2001a, b; Vane 2003) and spectra from synthetic phenol standards (Table 1). Phenols were quantified using the internal standard approach, then normalised to 100 mg OC using the sample TOC determined as described above. Total phenol concentrations were determined as the sum of the organic carbon normalised concentrations of the individual phenols detected.

**Table 1:** Phenols (with abbreviations) identified in litter, soils and leachates using thermally assisted hydrolysis and methylation on in-line pyrolysis gas chromatography mass spectrometry (Py-GC-MS)

Abbreviation	Compound	Mass ions (m/z)
P1	Methoxybenzene	65, 77, 108
P2	4-methoxytoluene	91, 107, 122
G1	1,2-dimethoxybenzene	95,123, 138
P3	4-methoxybenzeneethylene	91, 119, 134
G2	3,4-dimethoxytoluene	109, 137, 152
S1	1,2,3-trimethoxybenzene	110, 153, 168
P24	4-methoxybenzeneacetic acid	91, 121, 180
G3	3,4-dimethoxybenzeneethylene	121, 149, 164
P6	4-methoxybenzoic acid methyl ester	77, 135, 166
S2	3,4,5-trimethoxytoluene	139, 167, 182
G4	3,4-dimethoxybenzaldehyde	151, 165, 166
G21	1-(3,4-dimethoxyphenyl)-3-propene	147, 163, 178
G5	3,4-dimethoxyacetophenone	137, 165, 180
G6	3,4-dimethoxybenzoic acid methyl ester	165, 181, 196
S4	3,4,5-trimethoxybenzaldehyde	125, 181, 196
G24	3,4-dimethoxybenzeneacetic acid methyl ester	107, 151, 210
S21	1-(3,4,5-trimethoxyphenyl)-3-propene	
P18	2-Propenoic acid, 3-(4-methoxyphenyl) methyl ester (E)-	133, 161, 192
S5	3,4,5-trimethoxyacetophenone	139, 195, 210
S6	3,4,5-trimethoxybenzoic acid methyl ester	195, 211, 226
G18	2-propenoic acid, 3-(3,4-dimethoxyphenyl) methyl ester	191, 207, 222

### Statistical analysis

GenStat (Release 14.1, VSN International Ltd., Hemel Hempstead, UK) was used to analyse all data using correlation, PCA and Analysis of variance (ANOVA) and specific differences were determined using the Fisher’s protected least significant difference (FPLSD) test. A two-sample t-test was used to compare differences between the following parameters in litter vegetation: litter % mass loss, % TOC mass loss, % TN mass loss, and % phenol mass loss, within the same leaf litter type. Replication was 4 for vegetation and soil analysis and 3 for leachate analysis. Statistical significance was tested at the 95 % level.

## Results

### Litter

Dry matter was greater in the leaves of the woody species oak and ash compared with the non-woody grasses and buttercup (Table 2). Mass loss after 671 days was in the order buttercup > ash > grass > oak. Total organic carbon ranged from 49.1 % in oak litter to 40.5 % in buttercup litter initially, which was reduced to 43.9 % (oak) and 33.3 % (buttercup) at 671 days. The greatest TOC losses were observed in grass (6.8 % loss) and the smallest in ash (5.4 % loss). Total nitrogen was significantly greater in buttercup (4.1 %) than in the other litters which ranged from 1.4 % (ash) to 1.9 % (grass), and TN losses after 671 days were very small or not detectable in any litter type. The largest changes in C:N ratios were found in the woody species because of the larger relative TOC losses.

**Table 2:** Soil and litter (grass, buttercup, ash, and oak) characteristics at the beginning (0 days) and end (671 days) of the lysimeter experiment. Values are mean (*n* = 4) ± 1 s.e.

Days		Soil (control)	Grass	Buttercup	Ash	Oak
		% dry weight				
0	Dry matter	–	16.3 (1.1)	12.6 (0.5)	22.1 (1.3)	25.5 (1.0)
	TOC	1.3 (0.19)	43.8 (0.46)	40.5 (0.53)	47.1 (0.18)	49.1 (0.28)
	TN	0.1 (0.00)	1.9 (0.10)	4.1 (0.22)	1.4 (0.04)	1.6 (0.05)
	C:N	13	23	10	34	31
	Phenols:N	–	0.31	0.07	0.43	0.41
671	Mass loss	–	38.9 (0.6)	83.4 (4.6)	42.9 (2.5)	22.7 (0.7)
	TOC	0.6 (0.14)	37.0 (0.85)	33.8 (2.41)	41.7 (0.90)	43.9 (1.14)
	TN	0.1 (0.01)	1.9 (0.06)	3.9 (0.33)	1.7 (0.06)	1.7 (0.05)
	C:N	6	19	9	25	26
	Phenols:N	–	0.38	0.12	0.35	0.37

**Fig. 1:**
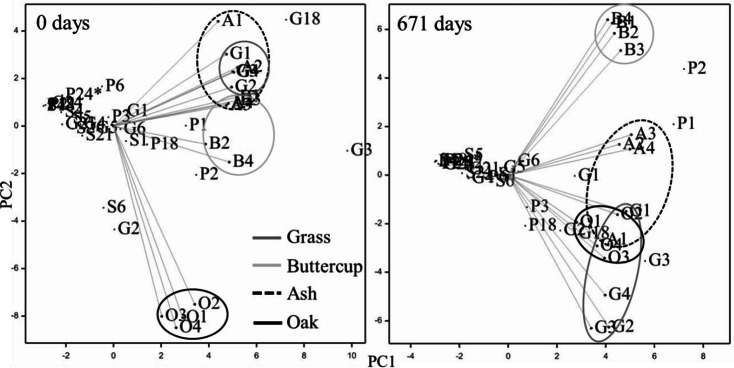
Principal component analysis of first and second components of individual phenol concentrations (abbreviation definitions given in Table 1), and variates: grass (G), buttercup (B), ash (A), and oak (O) litter samples. Numbers 1 to 4 identify replicates

There was a trend for increased (up to double) total phenol concentrations in all degraded litter types (Table 3), where the greatest proportional increase was observed in buttercup (651 to 1384 μg/100 mg OC). Initial phenol:N ratios were greatest in woody litter types (0.43 and 0.41, for ash and oak, respectively), and least in buttercup (0.07; Table 2). The loss of total phenols was 74 % (buttercup), 40 % (ash), 29 % (oak), and 24 % (grass) of the initial concentration, but there were no significant differences between litter types because of the large variation in concentration between individual phenols. After 671 days, phenol:N ratios had increased in the herbaceous species, but decreased in the woody species. Some relative differences in individual phenol concentrations between litter types were observed, but these were different in the initial litter (0 days) compared to the degraded litter (671 days; Fig. 1). There was a trend for the relative abundances of phenols to increase that was more pronounced in the non-woody litter types (Table 2). For example, the initial oak litter was different from the other litter types based on the greater relative abundance of S6 (184.5 ± 61.8 μg 100 mg OC) and G2 (162.6 ± 13.8 μg 100 mg OC) phenols in particular (Fig. 1; Table 3). After 671 days, there was no distinction between grass, ash and oak litter, but the buttercup litter separated in the PCA plot based on the relative abundance of P2 (411.1 ± 184.6 μg 100 mg OC) and G6 (101.8 ± 51.3 μg 100 mg OC) phenols, and relatively lower abundance of G2 (9.5 ± 9.5 μg 100 mg OC) and G3 (62.0 ± 20.7 μg 100 mg OC) compared with the other litter types (Fig. 1b; Table 3).

**Table 3:** Mean (± 1 s.e.; *n* = 4) normalised (μg/100 mg OC) phenol concentrations from TMAH/py-GC-MS analysis of grass, buttercup, ash and oak litters after 0 and 671 days. Total phenols (sum of all phenols) and acid/aldehyde ratios: G6/G4 and S6/S4 are also reported

Days	0				671			
	Grass	Buttercup	Ash	Oak	Grass	Buttercup	Ash	Oak
Compound	μg/100 mg OC							
P1	75.5 (14.8)	80.8 (19.7)	81.8 (8.0)	57.5 (5.9)	158.2 (29.5)	298.5 (134.5)	168.1 (32.0)	141.1 (21.2)
P2	72.1 (9.3)	93.8 (26.8)	67.8 (11.0)	104.3 (5.0)	134.2 (20.8)	411.1 (184.6)	196.2 (43.7)	97.6 (11.6)
G1	27.8 (4.3)	59.9 (18.7)	58.3 (11.3)	22.1 (11.9)	120.7 (24.1)	79.1 (19.1)	129.0 (49.1)	102.2 (44.1)
P3	69.2 (6.7)	18.4 (3.7)	44.8 (16.0)	40.3 (10.4)	127.9 (31.6)	28.6 (8.2)	66.3 (10.7)	76.3 (26.2)
G2	20.7 (4.9)	11.3 (11.3)	33.8 (8.1)	162.6 (13.8)	102.3 (19.7)	9.5 (9.5)	69.2 (18.8)	202.2 (29.0)
S1	35.8 (6.1)	48.2 (14.5)	50.2 (15.1)	64.5 (2.8)	41.7 (20.3)	21.1 (10.4)	45.8 (16.8)	82.0 (38.1)
P24	n.d.	n.d.	63.5 (38.3)	n.d.	n.d.	n.d.	25.2 (12.7)	n.d.
G3	280.5 (54.0)	122.3 (40.6)	215.7 (60.4)	166.2 (17.6)	293.3 (31.1)	62.0 (20.7)	139.9 (40.4)	173.4 (28.3)
P6	98.4 (23.8)	24.4 (6.6)	26.9 (10.5)	n.d.	96.7 (30.6)	66.1 (37.2)	15.0 (2.7)	14.4 (3.0)
S2	11.7 (4.7)	17.1 (5.6)	10.7 (4.4)	33.2 (10.3)	15.9 (6.3)	12.9 (5.4)	24.9 (9.5)	25.7 (9.4)
G4	31.0 (3.1)	6.1 (3.6)	33.8 (11.6)	39.2 (9.3)	42.7 (13.4)	5.9 (3.4)	38.1 (20.5)	28.1 (11.4)
G21	8.9 (3.6)	n.d.	18.5 (3.8)	29.5 (5.2)	20.5 (3.5)	7.2 (5.3)	33.0 (10.9)	37.7 (18.1)
G5	37.7 (11.2)	n.d.	63.4 (18.3)	74.4 (37.5)	39.1 (1.1)	73.5 (37.8)	31.7 (11.5)	82.6 (6.8)
G6	30.7 (5.8)	44.7 (14.1)	62.2 (24.3)	56.6 (15.2)	62.3 (8.5)	101.8 (51.3)	59.0 (18.8)	58.5 (16.4)
S4	13.8 (1.9)	n.d.	28.1 (14.6)	18.6 (6.2)	n.d.	n.d.	1.9 (1.9)	5.1 (3.0)
G24	n.d.	n.d.	15.2 (8.3)	n.d.	4.5 (4.5)	n.d.	2.0 (1.3)	12.6 (2.9)
S21	2.2 (0.817)	n.d.	51.4 (21.6)	49.0 (5.1)	9.9 (5.7)	n.d.	25.8 (8.9)	46.7 (12.6)
P18	176.0 (16.1)	8.4 (3.4)	43.7 (16.0)	101.0 (34.8)	183.7 (42.6)	28.4 (9.6)	50.4 (14.5)	51.5 (7.3)
S5	24.3 (2.7)	10.0 (2.3)	13.4 (7.0)	27.5 (1.6)	27.9 (10.4)	54.7 (33.1)	14.6 (3.3)	8.2 (1.95)
S6	36.4 (7.7)	9.8 (2.7)	20.2 (12.3)	184.5 (61.8)	53.4 (20.6)	65.1 (40.8)	17.9 (8.6)	79.2 (23.8)
G18	290.6 (43.7)	66.2 (12.4)	273.6 (127.8)	14.3 (2.1)	233.7 (62.1)	51.2 (26.1)	205.0 (96.3)	11.1 (2.0)
Total phenols	1366 (189)	651 (171)	1303 (379)	1297 (202)	1909 (151)	1384 (550)	1421 (368)	1450 (172)

*n.d.* not detected

### Soil

The control soil TOC was reduced from 1.3 to 0.6 % after 671 days (Table 2). There was no detectable change in TN. The detectable total phenol concentration of the soil was 60 ± 22 μg/100 mg OC. The detectable phenols were P1 (19 ± 8 μg/100 mg OC), P2 (10 ± 7 μg/100 mg OC), P6 (18 ± 18 μg/100 mg OC), G6 (8 ± 8 μg/100 mg OC) and S6 (19 ± 8 μg/100 mg OC).

### Leachates

#### Dissolved organic carbon

**Fig. 2:**
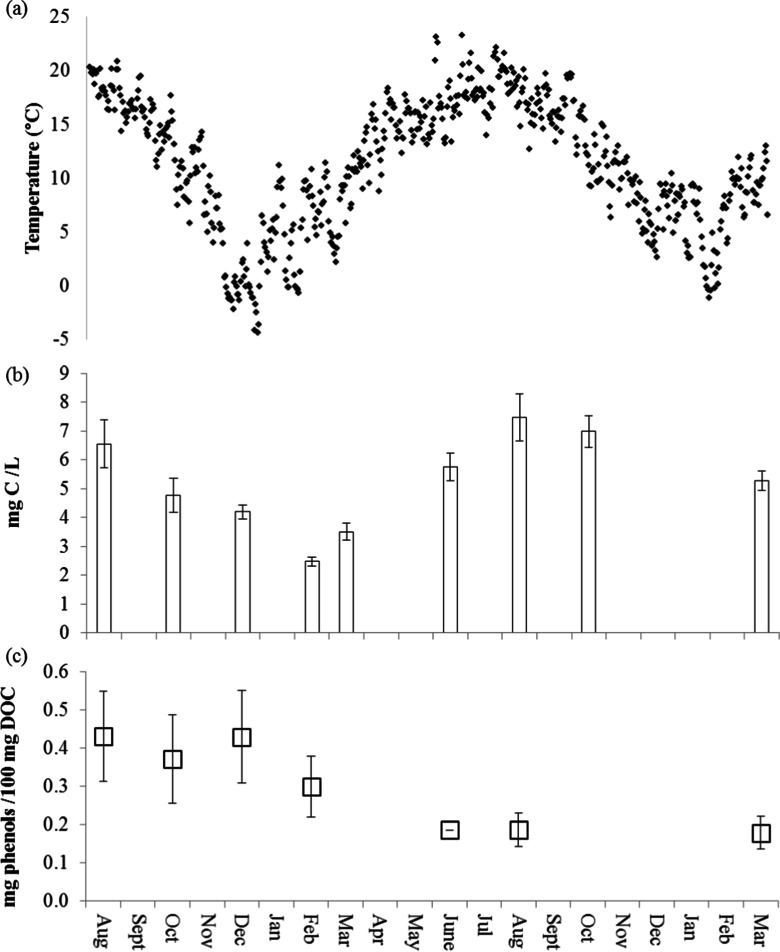
The relationship between **a** soil temperature and mean (*n* = 3) **b** dissolved organic carbon concentrations (mg C/L) and **c** total phenol concentrations in leachates collected from control (no litter) soil lysimeters after 82, 143, 200, 263, 381, 459, and 671 days. *Error bars* indicate ±1 s.e

**Fig. 3:**
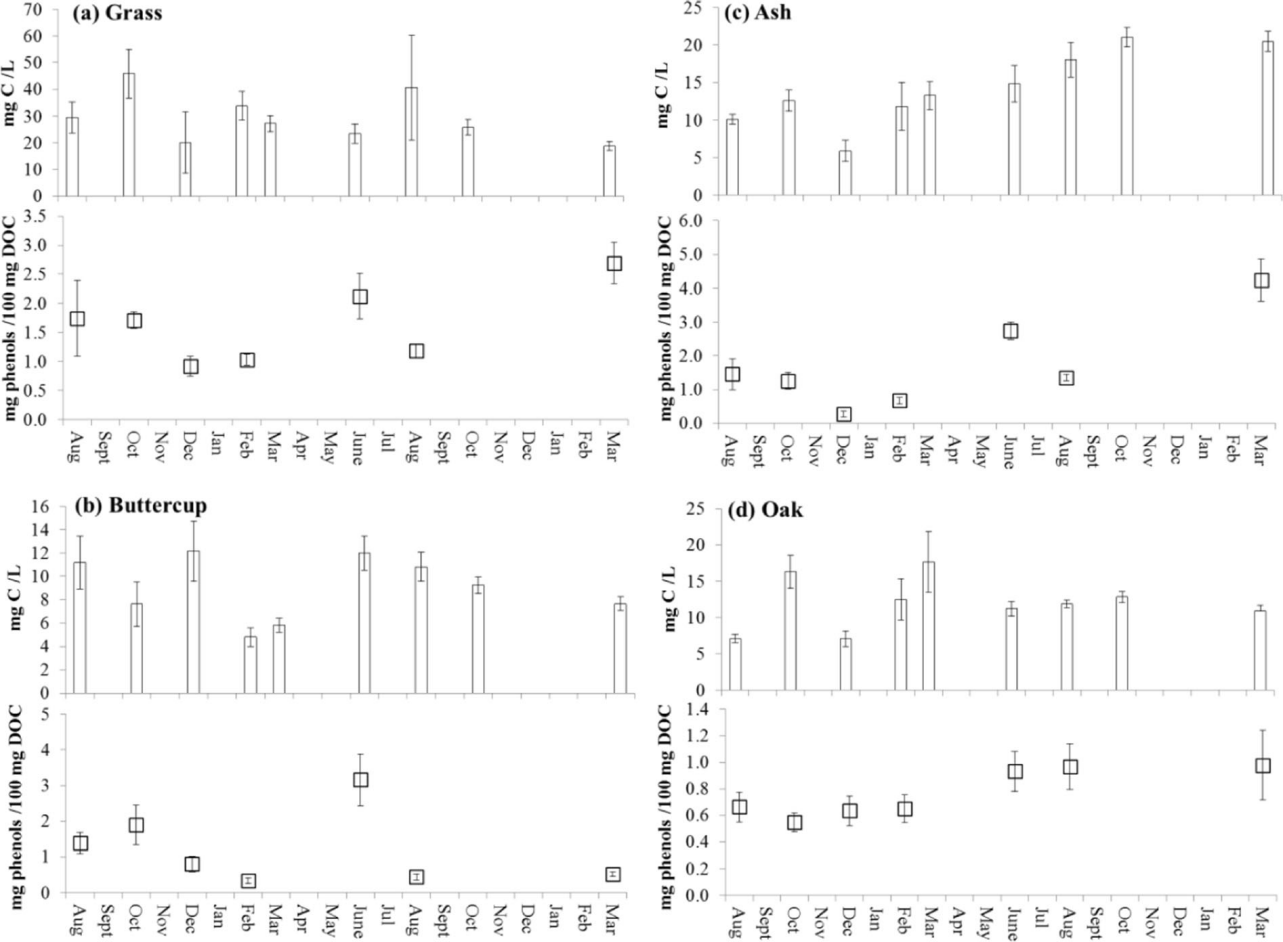
Mean (*n* = 3) dissolved organic carbon concentrations (mg C/L) and total phenol concentrations (μg/100 mg C) in leachates collected from soil lysimeters treated with **a** grass, **b** buttercup, **c** ash and **d** oak litters after 82, 143, 200, 263, 381, 459, and 671 days. *Error bars* indicate ±1 s.e

There was a significant positive correlation (r^2^ = 0.6917) between soil temperature change (annual mean 11 °C; summer maximum 34 °C; winter minimum −7 °C; Fig. 2a) and corresponding maximum DOC concentrations in leachates from the control soil in the summer of year 1 (82 days; 6.55 mg C/L) and year 2 (459 days; 7.47 mg C/L) and minima in winter (263 days; 2.47 mg C/L; Fig. 2b). Correlations between soil temperature change and DOC concentrations were weaker in litter treated soils (Fig. 3; r^2^ = 0.1331, 0.1023, 0.2470, and 0.0335 for grass, buttercup, ash and oak treatments, respectively) because the different litters exhibited different trends. DOC in leachates from ash litter-treated soil increased over time (Fig. 3c), whereas those for grass (Fig. 3a) and buttercup (Fig. 3b) leachates showed a trend to decrease through time, and fluctuated before reaching a stable concentration in oak leachates (Fig. 3d). Concentrations of DOC in leachates differed between plant litter treatments at different times of the year. Maximum OC measured in leachates differed between litter treatments in the order: grass litter 45.89 ± 9.02 mg C/L (143 days; Fig. 3a) > ash litter 21.04 ± 1.26 mg C/L in (523 days; Fig. 3c) > oak litter 17.66 ± 4.22 mg C/L (312 days; Fig. 3d) > buttercup litter 12.16 ± 2.57 mg C/L (200 days; Fig. 3b).

#### Total phenols in leachates

Unlike the DOC concentrations, the DOC normalised total phenol concentrations in leachates from the control treatment did not correlate with temperature (r^2^ = 0.1362) but reduced progressively with time from 0.43 ± 0.01 μg/100 mg OC (82 days) to 0.18 ± 0.00 μg/100 mg (671 days; Fig. 2c). Total phenol concentrations in leachates from the litter-treated soils were always greater than the control treatment, and correlations with soil temperature change over the duration of the experiment were: r^2^ = 0.1549, 0.1977, 0.1218, and 0.1877 for grass, buttercup, ash and oak litters, respectively (Fig. 3a, b, and c). However, there was a significant positive correlation between the release of total phenols in leachates from the grass treatment and seasonal temperature until June in the second year (R^2^ = 0.9069, *P* < 0.02) and a significant positive correlation between total phenols leached from the ash treatment and temperature until February in the second year (R^2^ = 0.9838, *P* < 0.02). The correlation between phenol concentration and temperature was weaker in buttercup leachates (R^2^ = 0.5628 until June in the second year), and no correlation was observed in the leachates of the oak litter treatment where the total phenolic content increased progressively with time from 0.66 to 0.98 μg/100 mg OC (Fig. 3d). The maximum total phenol concentrations differed between litter treatments in the order: ash litter (4.24 ± 0.04 μg/100 mg OC; 671 days) > buttercup litter (3.16 ± 0.04 μg/100 mg OC; 381 days) > grass litter (2.69 ± 0.03 μg/100 mg OC; 671 days) > oak litter (0.98 ± 0.02 μg/100 mg OC; 671 days).

#### Individual phenols in leachates

**Fig. 4:**
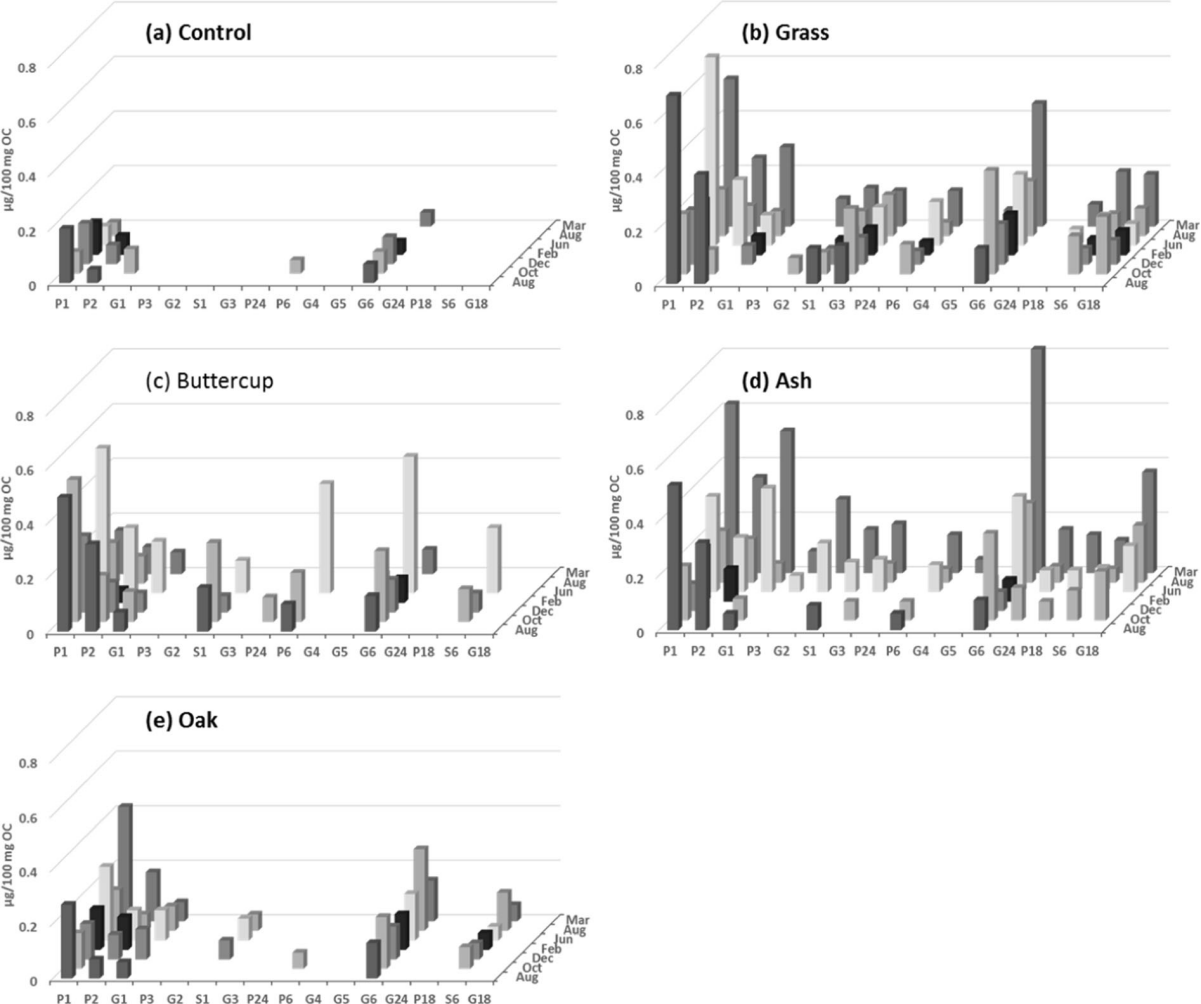
Mean concentrations of phenols in leachates from soils treated with **a** no litter (control), **b** grass litter, **c** buttercup litter, **d** ash litter and **e**s oak litter after 82, 143, 200, 263, 381, 459, and 671 days. (Mean values and standard errors given in Supplementary Table 2)

The concentrations of individual phenols (with concentrations greater than 0.05 μg/100 mg OC) in the leachates from the control treatment remained relatively constant through time, except P1 which decreased (0.20 to 0.05 μg/100 mg OC, Fig. 4, Supplementary Table 2). Individual phenols in leachates from the soils treated with grass, buttercup, and ash litter types were similar in identity, and the variety of phenols increased with time and was most diverse at peak total phenol concentrations, in June (381 days) and March (671 days) (Fig. 3a, b, c). For example, P1 was detected in all leachates in relatively high abundances throughout the incubation, i.e. after 82 days in grass (0.69 ± 0.53 μg/100 mg OC), buttercup (0.49 ± 0.21 μg/100 mg OC), ash (0.53 ± 0.41 μg/100 mg OC) and oak (0.27 ± 0.09 μg/100 mg OC) litter treatments, and after 671 days in grass (0.54 ± 0.10 μg/100 mg OC) and ash (0.62 ± 0.21 μg/100 mg OC) but not buttercup litter treatments where concentrations had declined to 0.16 ± 0.05 μg/100 mg OC at 671 days from a maximum of 0.53 ± 0.37 μg/100 mg OC at 381 days. Greater concentrations of G6 were associated with greater concentrations of S6 in leachates of all litter types in the latter half of the experiment. G6 was the most abundant phenol in leachates from ash litter treatments determined at 0.82 ± 0.26 μg/100 mg OC and second most abundant phenol from grass litter treatments (0.45 ± 0.21 μg/100 mg OC) after 671 days, respectively; 0.50 ± 0.36 μg/100 mg OC after 381 days for the buttercup litter treatment; and, 0.30 ± 0.13 μg/100 mg OC after 459 days in the oak litter treatment. P3 was only detected >0.05 μg/100 mg OC after 381 days in the leachates from the ash treatment. P24 and G24 were only identified in the leachates (>0.05 μg/100 mg OC) from the buttercup and ash treatments, respectively.

## Discussion

### Litter degradation varied between plant types

Using senescent leaves of four dominant local plant types as the treatment in this study, we observed the relationship between plant type and decomposition as: buttercup > ash > grass > oak. This agrees with a recent meta-analysis of the published global data by Cornwell et al. (2008) relating to the relationships between leaf litter traits and decomposition rates, who observed that the litter from graminoid species did not necessarily decompose faster than woody species litter. The woody species used in this study were likely to have different decomposition rates; Riutta et al. (2012) described ash as ‘easily decomposable’ and oak as ‘decomposition resistant’ and observed that leaves from freshly fallen ash and oak leaves lost 87 % and 57 % mass, respectively, in a 3 month litter incubation in UK woodland. Buttercup litter decomposed fastest (83 % in 671 days), and had the lowest C:N ratio because it contained two- to three-times more N than the other litter types, suggesting a relationship between litter N and decomposition rate. The reduced availability of N may inhibit decomposition where N supply is limited, e.g. in high C:N litters (e.g. wheat straw; Mary et al. 1996) or increase it because the activity of fungi which can degrade lignin to CO_2_ are repressed by high N substrates (Berg and McClaugherty 2014). However, the proportion of N in all litter types did not change during the experiment, even in the buttercup, suggesting that easily available N was lost before the litter was collected, similar to that reported by Sanaullah et al. (2010) for ‘brown’ litters. This indicates that the N remaining in the senesced litters was in a form that was resistant to decomposition, i.e. through the formation of polyphenol-protein condensates via the reaction of carbonyl (C=O) groups in [partially oxidised] lignin with NH_2_ groups to form Schiff bases, or complexes of tannins with proteins via ionic and hydrogen bonding or hydrophobic interactions that are assumed to resist microbial degradation (see the review by Knicker 2011). The oak litter had the slowest rate of decomposition (23 % mass loss) of the four plant types which may be ascribed to the larger relative abundance of tannins in this litter type (Filley et al. 2006). However, the source of the litter N that was measured was not defined, so it cannot be ruled out that a proportion may have been derived from the colonising decomposers; Zeller et al. (2000) reported that up to 35 % of litter N was microbial after 3 years.

Lignin is widely reported to account for a large percentage of the dry weight of leaves and leaf litter, e.g. 10 to 24 % of fresh leaves from a range of broad-leaved deciduous trees (Melillo et al. 1982); 28 to 51 % of fresh leaf litter of coniferous and broad-leaved deciduous trees (Berg et al. 2007); and, 3, 4 and 10 % of senescent cereal straw, pasture grass and native woodland litter, respectively (Walela et al. 2014). However, the gravimetric analytical methods used in many studies (including those listed above) to ‘quantify’ lignin may lead to a substantial overestimation because the ‘lignin fraction’ includes a range of non-lignin substances that are resistant to strong acids or detergents. A recent study by Klotzbücher et al. (2011a) compared three widely-used methods for lignin analysis and determined very large differences between the lignin content estimated for a range of tree leaves, e.g. ~2, ~5 and ~50 % litter C, respectively, from ^13^C-TMAH thermochemolysis, CuO oxidation and acid detergent lignin (ADL) analyses of ash leaves that had been incubated for 3 months. Although an underestimation of lignin using the former two methods is suspected (because of the preferential cleavage of specific bonds by either method), there is a difference of an order of magnitude compared with the results of ADL. The application of TMAH in this experiment to the initial senesced litters detected total phenols ranging from <1.0 % (buttercup) to 1.4 % (grass) litter C, and between 1.4 % (buttercup) and 2.0 % (grass) litter C after 671 days (Table 3). This relatively minor contribution to leaf dry matter in senesced leaves concurs with the emerging idea put forward by Klotzbücher et al. (2011b), that lignin degradation in the early stages of litter decomposition is pronounced with the majority lost as CO_2,_ and that lignin input to soil (and therefore lost in leachates) is actually rather minor.

Chabbi and Rumpel (2004) and Sanaullah et al. (2012) reported that during the first phase (<11 months) of decomposition plant material is degraded as a whole, rather than selectively, and during the second phase (>11 months) more readily degradable components such as polysaccharides are selectively degraded. The stage of decomposition of the senescing litters used in this experiment was unknown. However, as previously reported for decomposing grasses (Sanaullah et al. 2010; Bray et al. 2012) and tree leaves (Klotzbücher et al. 2011a) we observed an increase in the proportion of total phenols remaining in all litter types after 671 days. Furthermore, the contributing phenols had changed in relative abundance (Fig. 1), suggesting that different monomers were released from the leaf litter at different rates in different plant types. Moreover, we investigated phenol decomposition from single species in isolation over a time course extending beyond 1 year. It is acknowledged that in vivo each individual species and coexisting vegetation would contribute more litter, and also that natural populations of litter and soil meso- and macrofauna can considerably alter litter decomposition rates, despite unaltered climatic conditions and litter chemistry (Hattenschwiler and Gasser 2005). Furthermore, the ramification of the litter by fungal mycelia which decompose lignin specifically (Robertson et al. 2008) and increase DOC production (Kalbitz et al. 2000) were likely excluded from this study by experimental sampling preparation and design. The use of grassland soils for the experiment may have also conferred ‘home field advantage’ on the grass and buttercup treatments wherein the soil microbial community was already adapted to this litter type (Austin et al. 2014), and was potentially more compatible with the microbial community in the senescing grass and buttercup litter than with that in the ash and oak litter.

### Effect of seasonal temperature change on DOC loss from soils

Future temperature increases will change SOC decomposition patterns by affecting soil microbial community dynamics and substrate utilization (Pisani et al. 2015), and temperature-dependant processes affecting the release of DOC from soils, e.g. sorption or immobilisation that are controlled by microbial activity (Marschner and Bredow 2002) or physicochemical factors, e.g. dissolution, diffusion and exchange reactions (Toosi et al. 2014). Peak DOC concentrations in soils are generally observed during the summer due to increased inputs from root exudates and the elevated activity of decomposers in plant litter and soil, and (in temperate and tropical regions) enhanced by the effect of wet-dry cycles which have physical impacts on soil function and biological activity (Placella et al. 2012). In the control soils, in the absence of plant litter, a significant positive relationship between mean soil temperature and the concentrations of DOC leached from the lysimeters was detected which we assume was the direct impact of temperature on the soil microbial biomass, often observed as changes in respiration in similar experiments using unplanted, packed soils e.g. Karhu et al. (2014). More than half the initial TOC concentration was lost in the control soil by the end of the experiment (0.6 %), but we did not observe a coincident reduction in the loss of DOC over time. This suggests that the processes controlling the release of DOC were not limited by the biological availability of SOC within the timescale of the experiment, i.e. that the SOC was sufficiently abundant and bioavailable to permit the observation of seasonal DOC trends. Alternative mechanisms for the supply of SOC that rely on its abiotic release from stabilised SOC have been hypothesised but are difficult to test experimentally due to the challenge to separate biotic from abiotic mechanisms in soils (Kemmitt et al. 2008; Paterson 2009; Toosi et al. 2012).

The DOC leached from soil was very small; therefore, we assume that the vast majority of applied litter-derived OC either remained in the soil or was oxidised to CO_2_ (as observed by Klotzbücher et al. 2011b). Nevertheless, the DOC losses were much larger from the litter-treated soils, but no correlation was observed between temperature and DOC loss. Different patterns of DOC loss were determined between different litter types, e.g. an increased leaching of DOC over time in the ash treatment from December in year 1 compared to a stable rate of loss from the oak treatment after June in year 2. The explanation for the differences is very difficult to define and must incorporate a range of physicochemical and biological mechanisms in the litter and soils. The purely physical transport of largely unmodified litter-derived water transportable organic matter through the soil matrix as soluble compounds in leachates or colloids, modified by adsorption/desorption (Kaiser and Kalbitz 2012), could explain the increase in DOC from the treated lysimeters. The source of the carbon (litter or SOC) in DOC has been investigated using stable (^13^C) or radio (^14^C)-isotopes to differentiate source (Kahl et al. 2012; Tipping et al. 2012; Toosi et al. 2012; Scheibe and Gleixner 2014), so compound-specific stable isotope analyses of ^13^C-labelled phenols could help to reveal rates of transport, transformation and loss in experimental systems in future studies. The less tangible effects are those of the litter-derived organic inputs on the soil microbial community. The biodegradability of the DOC, and therefore its effect on soil microorganisms and its potential for decomposition, depends on the biochemistry of the component molecules and their relative abundances (Kalbitz et al. 2003; Dungait et al. 2011, 2013). The leached DOC from the plant litter may have accelerated the loss of SOC by positive priming of the soil microbial biomass, i.e. supplying limiting C or nutrients that allowed the proliferation of the microbial community and the subsequent increased decomposition and loss of SOC (Kuzyakov 2010). Microbial activity can also modify soil physical structure (Stockmann et al. 2013) and therefore the hydrological pathways through soils altering the potential for physical transport or stabilisation of DOC.

### Effect of seasonal temperature change on phenol loss from soils

The contribution of plant-derived phenols to the small amount of DOC lost from the soil lysimeters was consequently diminutive, ranging between 0.8 ± 0.1 (oak) and 1.7 ± 0.3 μg/100 mg OC (ash) during the 671 day incubation. Total phenol concentrations in leachates from the control treatment (i.e. no litter) tended to reduce progressively with time, unlike the DOC concentrations which correlated positively with temperature change (Fig. 2). This suggests that there are processes controlling the rate of release of dissolved phenols from SOC which are temperature independent. Lignin phenols are one of the most reactive plant-derived compounds towards soil mineral surfaces and up to 56 % of sorbed lignin can be irreversibly bound to minerals where individual monomers and their conformations may have different sorption bond strengths (Hernes et al. 2013) and therefore may not display obvious seasonal temperature trends. Phenolic compounds may also have anti-microbial activity (Balasundram et al. 2006) and bind proteins including free enzymes in soils (Freeman et al. 2004; Joanisse et al. 2007), both potentially diminishing decomposition.

In contrast to the bare soil controls, patterns of total phenol losses in the grass and ash litter treatments correlated positively with soil temperature dynamics in the first year (Fig. 3). Elevated ecosystem respiration rates in summer that coincide with peak activity of the soil microbial community (e.g. Kirschbaum 2013) and the effect of temperature on increasing desorption relative to adsorption rates (Le Chatelier’s principle, Conant et al. 2011) may combine to effect increased rates of DOC release. Kaiser et al. (2001) also reported increased concentrations of lignin-derived phenols in forest floor leachates in warm and moist conditions in summer and autumn conditions. However, the weaker relationship between temperature and phenol release from the buttercup and oak litter treatments suggest that additional processes contributed to the dynamics of phenol release from these litter types. The most obvious explanation is the effect of tannins on microbial activity, but our analytical approach did not allow the source of phenols to be differentiated. However, senesced oak leaves in particular are likely to contain elevated concentrations of tannins (Nierop and Filley 2007). We detected greater concentrations of S6 in oak compared to the other litter types, which could be lignin or tannin-derived and distinguishable using ^13^C-labelled TMAH (Filley et al. 2006).

## Conclusions

Plant-derived polyphenolic polymers such as lignin are a major input to the terrestrial C cycle that might be expected to contribute substantially to losses of DOC from soils. However, we observed that the overall phenol content of ‘brown’ senescing litters was not large and, consequently, that losses of phenols to water were also minor. During litter decomposition, the relative proportion of the phenol content to TOC increased with time in all litters except buttercup, and the concentration of different phenols changed between litters, suggesting that the individual phenolic composition of leaves contributes to the control of degradation rates. Similarly, the relative abundance and type of phenols lost as DOC changed over time in different plant litters. A strong relationship between seasonal temperature change and loss of DOC from the control soil (no litter) was observed, but this was either absent or only observed in the first year when the soils were treated with plant litter. A difference in phenol dynamics in DOC between plant litter types was determined, suggesting that phenol chemistry plays a role in controlling losses to water.

## Electronic supplementary material

Supplementary Table 1 (DOCX 12 kb)

Supplementary Table 2 (DOCX 15 kb)
