# Supplementary material for: Contrasting temperature responses of dissolved organic carbon and phenols leached from soils
Source: Plant Soil. 2015 Sep 23;399:13–27. doi: 10.1007/s11104-015-2678-z (PMC4750429; doi:10.1007/s11104-015-2678-z)
Supplement: Supplementary file 1 — (DOCX 12 kb) [file 11104_2015_2678_MOESM1_ESM.docx]

Supplementary Table 1: Mean (n = 4; ± s.e.) of pH values of leachates from lysimeters for each sample date under each litter treatment.

|  | Control | | Grass | | Buttercup | | Ash | | Oak | |
| --- | --- | --- | --- | --- | --- | --- | --- | --- | --- | --- |
| Sampling date | pH | s.e. | pH | s.e. | pH | s.e. | pH | s.e. | pH | s.e. |
| 10/08/2010 | 7.67 | 0.03 | 7.27 | 0.09 | 7.62 | 0.02 | 7.38 | 0.24 | 7.51 | 0.04 |
| 11/10/2010 | 7.68 | 0.02 | 7.19 | 0.08 | 7.59 | 0.01 | 7.66 | 0.04 | 7.49 | 0.02 |
| 06/12/2010 | 7.95 | 0.20 | 7.71 | 0.03 | 7.63 | 0.03 | 7.97 | 0.05 | 7.89 | 0.04 |
| 08/02/2011 | 7.82 | 0.03 | 7.59 | 0.05 | 7.74 | 0.03 | 7.82 | 0.02 | 7.74 | 0.03 |
| 28/03/2011 | 7.90 | 0.02 | 7.54 | 0.04 | 7.76 | 0.02 | 7.86 | 0.04 | 7.71 | 0.03 |
| 06/06/2011 | 7.75 | 0.03 | 7.52 | 0.08 | 7.68 | 0.04 | 7.70 | 0.03 | 7.61 | 0.06 |
| 22/08/2011 | 7.38 | 0.29 | 7.40 | 0.14 | 7.61 | 0.02 | 7.73 | 0.04 | 7.63 | 0.06 |
| 25/10/2011 | 7.70 | 0.03 | 7.54 | 0.05 | 7.57 | 0.05 | 7.80 | 0.02 | 7.65 | 0.07 |
| 21/03/2012 | 7.70 | 0.03 | 7.55 | 0.06 | 7.64 | 0.06 | 7.66 | 0.02 | 7.54 | 0.03 |
