# Supplementary material for: Contrasting temperature responses of dissolved organic carbon and phenols leached from soils
Source: Plant Soil. 2015 Sep 23;399:13–27. doi: 10.1007/s11104-015-2678-z (PMC4750429; doi:10.1007/s11104-015-2678-z)
Supplement: Supplementary file 2 — (DOCX 15 kb) [file 11104_2015_2678_MOESM2_ESM.docx]

Supplementary Table 2. Mean (n = 3, ± 1s.e.) concentrations of phenols (> 0.05 µg /100 mg DOC) in leachates from soils treated with (a) no litter (control), (b) grass litter, (c) buttercup litter, (d) ash litter and (e) oak litter after 82, 143, 200, 263, 381, 459, and 671 days.

| Days (month) | 82  (August) | 143  (October) | 200 (December) | 263  (February) | 381  (June) | 459 (August) | 671 (March) |
| --- | --- | --- | --- | --- | --- | --- | --- |
| Compound | µg /100 mg DOC | | | | | | |
| **(a) control** |  |  |  |  |  |  |  |
| P1 | 0.20 (0.11) | 0.08 (0.04) | 0.15 (0.10) | 0.12 (0.06) | 0.07 (0.00) | 0.05 (0.03) |  |
| P2 | 0.05 (0.02) |  | 0.07 (0.03) | 0.07 (0.04) |  |  |  |
| G1 |  | 0.09 (0.06) |  |  |  |  |  |
| P6 |  | 0.05 (0.05) |  |  |  |  |  |
| G6 | 0.07 (0.02) | 0.08 (0.06) | 0.10 (0.05) | 0.05 (0.02) |  |  | 0.05 (0.03) |
| **(b) grass** |  | | | | | | |
| P1 | 0.69 (0.53) | 0.22 (0.05) | 0.20 (0.10) | 0.20 (0.10) | 0.69 (0.30) | 0.17 (0.08) | 0.54 (0.10) |
| P2 | 0.40 (0.36) | 0.09 (0.03) |  |  | 0.24 (0.17) | 0.11 (0.04) | 0.25 (0.09) |
| G1 |  |  | 0.07 (0.04) | 0.07 (0.04) | 0.11 (0.08) | 0.09 (0.01) | 0.29 (0.15) |
| G2 |  | 0.06 (0.05) |  |  |  |  | 0.10 (0.09) |
| S1 | 0.13 (0.06) | 0.08 (0.04) | 0.06 (0.02) | 0.06 (0.02) | 0.06 (0.06) | 0.09 (0.02) | 0.14 (0.05) |
| G3 | 0.14 (0.05) | 0.24 (0.07) | 0.10 (0.08) | 0.10 (0.08) | 0.14 (0.09) | 0.15 (0.04) | 0.13 (0.05) |
| P6 |  | 0.11 (0.02) | 0.05 (0.02) | 0.05 (0.02) | 0.16 (0.03) | 0.05 (0.00) | 0.13 (0.06) |
| G5 |  |  |  |  |  |  | 0.06 (0.02) |
| G6 | 0.13 (0.07) | 0.38 (0.03) | 0.15 (0.07) | 0.15 (0.07) | 0.26 (0.06) | 0.20 (0.03) | 0.45 (0.21) |
| P18 |  |  |  |  | 0.06 (0.01) |  | 0.08 (0.03) |
| S6 |  | 0.14 (0.03) | 0.06 (0.02) | 0.06 (0.02) | 0.11 (0.02) | 0.08 (0.01) | 0.20 (0.09) |
| G18 |  | 0.21 (0.05) | 0.09 (0.07) | 0.09 (0.07) | 0.08 (0.03) | 0.10 (0.04) | 0.19 (0.09) |
| **(c) buttercup** |  | | | | | | |
| P1 | 0.49 (0.21) | 0.52 (0.38) | 0.28 (0.20) | 0.10 (0.07) | 0.53 (0.37) | 0.15 (0.07) | 0.16 (0.05) |
| P2 | 0.32 (0.17) | 0.17 (0.14) | 0.11 (0.04) | 0.05 (0.03) | 0.24 (0.11) | 0.10 (0.04) | 0.10 (0.04) |
| G1 | 0.07 (0.04) | 0.11 (0.07) | 0.07 (0.03) |  | 0.19 (0.11) |  | 0.08 (0.02) |
| S1 | 0.16 (0.11) | 0.29 (0.26) | 0.06 (0.05) |  | 0.12 (0.08) |  |  |
| P24 |  | 0.09 (0.09) |  |  |  |  |  |
| P6 | 0.10 (0.04) | 0.18 (0.11) |  |  | 0.40 (0.32) |  |  |
| G5 |  |  |  |  | 0.07 (0.03) |  |  |
| G6 | 0.13 (0.03) | 0.26 (0.16) | 0.12 (0.04) | 0.09 (0.03) | 0.50 (0.36) |  | 0.09 (0.01) |
| S6 |  | 0.12 (0.08) | 0.07(0.02) |  | 0.24 (0.17) |  |  |
| **(d) ash** |  | | | | | | |
| P1 | 0.53 (0.41) | 0.20 (0.08) | 0.10 (0.08) | 0.20 (0.05) | 0.35 (0.09) | 0.19 (0.04) | 0.62 (0.21) |
| P2 | 0.32 (0.21) |  |  | 0.12 (0.05) | 0.20 (0.05) | 0.16 (0.06) | 0.35 (0.24) |
| G1 | 0.06 (0.03) | 0.08 (0.01) |  |  | 0.38 (0.08) | 0.07 (0.01) | 0.52 (0.34) |
| P3 |  |  |  |  | 0.06 (0.03) |  | 0.08 (0.06) |
| G2 |  |  |  |  | 0.18 (0.06) |  | 0.27 (0.19) |
| S1 | 0.09 (0.06) |  |  |  | 0.11 (0.03) |  | 0.16 (0.11) |
| G3 |  | 0.07 (0.02) |  |  | 0.12 (0.05) | 0.07 (0.01) | 0.18 (0.13) |
| P6 | 0.06 (0.02) | 0.07 (0.02) |  |  | 0.10 (0.03) | 0.05 (0.01) | 0.14 (0.04) |
| G4 |  |  |  |  |  |  | 0.05 (0.04) |
| G5 |  |  |  |  | 0.05 (0.02) |  |  |
| G6 | 0.11 (0.04) | 0.32 (0.18) | 0.07 (0.04) | 0.08 (0.04) | 0.35 (0.09) | 0.29 (0.05) | 0.82 (0.26) |
| G24 |  | 0.12 (0.07) |  |  | 0.08 (0.02) | 0.06 (0.00) | 0.16 (0.04) |
| P18 |  | 0.07 (0.06) |  |  | 0.08 (0.03) |  | 0.14 (0.03) |
| S6 |  | 0.11 (0.06) |  |  | 0.09 (0.03) | 0.05 (0.01 | 0.12 (0.04) |
| G18 |  | 0.18 (0.08) |  |  | 0.17 (0.05) | 0.21 (0.03 | 0.37 (0.12) |
| **(e) oak** |  | | | | | | |
| P1 | 0.27 (0.09) | 0.13 (0.06) | 0.13 (0.06) | 0.15 (0.04) | 0.27 (0.10) | 0.15 (0.05) | 0.42 (0.23) |
| P2 | 0.07 (0.03) |  | 0.09 (0.03) | 0.12 (0.03) | 0.11 (0.06) | 0.06 (0.01) | 0.18 (0.06) |
| G1 | 0.06 (0.03) |  | 0.11 (0.05) |  | 0.11 (0.06) | 0.09 (0.04) | 0.07 (0.07) |
| S1 |  |  | 0.07 (0.06) |  | 0.08 (0.03) | 0.06 (0.02) |  |
| P6 |  | 0.06 (0.01) |  |  |  |  |  |
| G6 | 0.13 (0.05) | 0.19 (0.02) | 0.12 (0.04) | 0.13 (0.07) | 0.17 (0.05) | 0.30 (0.13) | 0.15 0.07) |
| S6 |  | 0.08 (0.01) | 0.06 (0.02) | 0.06 (0.04) | 0.05 (0.03) | 0.14 (0.08) | 0.06 (0.03) |
